# An Investigation of the Microstructure of an Intermetallic Layer in Welding Aluminum Alloys to Steel by MIG Process

**DOI:** 10.3390/ma8125444

**Published:** 2015-12-02

**Authors:** Quoc Manh Nguyen, Shyh-Chour Huang

**Affiliations:** Department of Mechanical Engineering, National Kaohsiung University of Applied Sciences, 415 Chien-Kung Road, Sanmin District, Kaohsiung 80778, Taiwan, ROC; manhrobocon@gmail.com

**Keywords:** dissimilar welding, A5052 alloy, SS400 steel, ER4043, intermetallic, butt-joint

## Abstract

Butt joints of A5052 aluminum alloy and SS400 steel, with a new type of chamfered edge, are welded by means of metal inert gas welding and ER4043 Al-Si filler metal. The microhardness and microstructure of the joint are investigated. An intermetallic layer is found on the surface of the welding seam and SS400 steel sheet. The hardness of the intermetallic layer is examined using the Vickers hardness test. The average hardness values at the Intermetallic (IMC) layer zone and without the IMC layer zone were higher than that of the welding wire ER4043. The tensile strength test showed a fracture at the intermetallic layer when the tensile strength is 225.9 MPa. The tensile value test indicated the average of welds was equivalent to the 85% tensile strength of the A5052 aluminum alloy. The thickness of the intermetallic layers is non-uniform at different positions with the ranges from 1.95 to 5 μm. The quality of the butt joint is better if the intermetallic layer is minimized. The Si crystals which appeared at the welding seam, indicating that this element participated actively during the welding process, also contributed to the IMC layer’s formation.

## 1. Introduction

Aluminum alloys are widely applied in the shipbuilding, aerospace, and automotive industries, as well as many other areas, because of their numerous advantages [1,2]. Steel, however, is used mostly in the manufacturing industry and in steel structures [3,4]. Combining these two materials can reduce both the time and cost of manufacturing, but it has been found that diffusion at the welds negatively affects weld quality. The chief difficulty is the considerable difference between the thermal-physical properties of aluminum alloys and steel. Intermetallic brittle and hard Fe*_x_*Al*_y_* easily occurs after welding, adversely affecting the welds. The level of diffusion of Fe in Al will result in the formation of the corresponding phase: Fe_3_Al, Fe_2_Al_5_, FeAl, FeAl_2_ or FeAl_3_. Various methods have been used to achieve optimal results when welding aluminum alloys and steel, such as friction stir welding [5,6], laser welding [2,7], resistance spot welding [8,9], and ultrasonic welding [10,11]. Metal inert gas (MIG) and metal active gas (MAG) welding is used as a welding heat source when welding steel and aluminum, and welding wire is provided semi-automatically throughout the process to fill the weld. The heat source causes highly non-uniform temperature distributions across the welding seam and the base metals. This welding process has five types of joints commonly used in industrial applications: butt, tee, corner, lap, and edge. Butt joint research is important because it deals with the complex problems of the welding process in the heat-affected zone (HAZ), specifically the bearing capacity and the reduction in quality due to the stress and distortion that always exist after welding. The aim of the present research is to investigate the butt joining of A5052 aluminum alloy and SS400 steel with a new type of chamfered edge using the MIG process.

## 2. Materials and Experimental Procedures

### 2.1. Materials

The materials used in this research include A5052 aluminum alloy sheets and SS400 steel sheets, with a thickness of 5 mm, and aluminum welding wire ER4043 (Al-5Si) with a diameter of 0.8 mm. The chemical compositions of the two base materials and the welding wire used in this research are shown in Table 1 [3], Table 2 [12], and Table 3 [13], respectively.

**Table 1:** Chemical composition of steel SS400 (wt %).

Material	Fe	C	Si	Mn	P	S
SS400	Bal.	0.16	0.16	0.67	0.014	0.006

**Table 2:** Chemical composition of A5052 aluminum alloys (wt %).

Material	Al	Zn	Si	Mn	Cu	Mg	Cr	Fe
A5052	Bal.	0.01	0.10	0.03	0.02	2.46	0.15	0.24

**Table 3:** Chemical composition of welding wire ER4043 (wt %).

Material	Si	Cu	Mg	Mn	Fe	Ti	Zn	Al
ER4043	4.5–6.0	0.3	0.05	0.05	0.8	0.20	0.1	Remain

**Figure 1:**
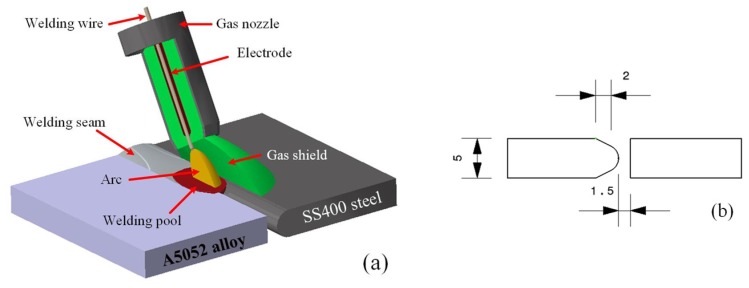
(**a**) Schematic of metal inert gas (MIG) welding process and (**b**) the shape of the new chamfer for A5052 aluminum alloy/SS400 steel.

### 2.2. Experimental Procedure

All sheets used in the experiments were cut to a size of 200 mm × 70 mm, and the surfaces of the base material sheets were cleaned with metal brushes and then with sandpaper before welding. The welding was carried out using an MIG/MAG pulsed welding source, and the welding parameters were a welding intensity of 85–100 A, a welding speed of 4 to 5 mm/s, a welding voltage in the range of 16–18 V, a contact tip to work distance (CTWD) of 8–10 mm, and an argon gas shield rate of 10–11 L/min. The schematic of the MIG welding process and the shape of the new chamfer between A5052 aluminum alloy and SS400 steel is shown in Figure 1. After the welding of 15 samples, seven were chosen for the tensile strength test. A typical cross-section of the specimens was cut and fixed in epoxy resin in a clamped condition. After that, the sample’s surface was polished to produce a mirror-like finish by the machine grinding of the specimen. The sample’s external form was inspected using an optical microscope (OM), and the Vickers microhardness test was performed on sections of the specimen. Finally, the microstructure of the IMC zones was analyzed by means of a scanning electron microscope (SEM, Jeol USA Inc., Peabody, MA, USA) and an energy dispersive X-ray spectrometer (EDS, Jeol USA Inc.).

## 3. Results and Discussion

**Figure 2:**
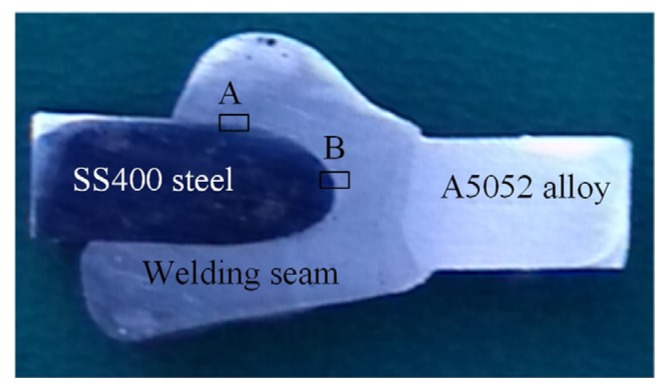
Macrographs of MIG welds between A5052 aluminum alloy and SS400 steel where A and B are micro-hardness testing IMC layer area and micro-hardness testing without IMC area, respectively.

Figure 2 shows the cross-section of the welding seam for pulse welding with a welding intensity of 95 A, a welding speed of 3.5–4 mm/s, a welding voltage of 16–18 V, a contact tip to work distance (CTWD) of 8–10 mm, and a gas shield flow rate of 10–11 L/min. The edge of the steel sheet had the new type of chamfered edge. The welding gap was about 2 to 3 mm because the wide welding gap on the top side helps the welding wire fuse evenly to the opposite (bottom) side, thereby limiting the welding defects when welding the bottom side, as well as improving the quality of the weld joint. At the A5052 aluminum alloy plate, the melting temperature was low, so the ER4043 welding wire and the sheets easily combined into a unity. At the SS400 steel surface, the melting temperature was higher; for the combination, it was less than that of the aluminum alloy sheets.

**Figure 3:**
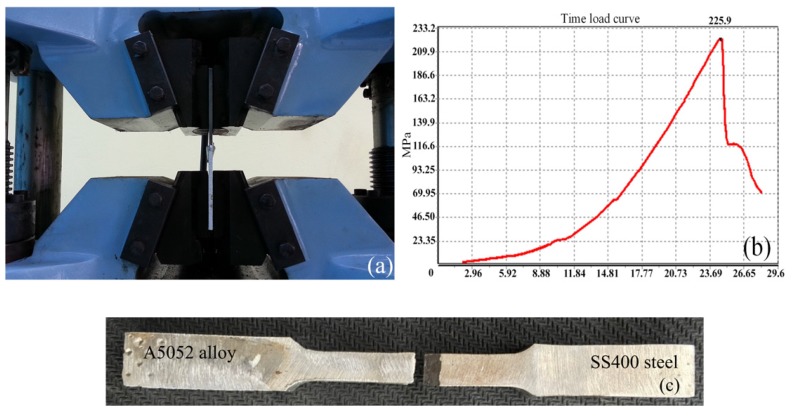
(**a**) Setup of tensile tests of welded samples; (**b**) Deformation load curve results; (**c**) Tensile specimens for metal inert gas (MIG) welding-brazing of A5052 alloy to SS400 steel.

An SHT4000 universal machine was used to test the weld’s tensibility. Seven samples were selected from the total of 15 welded samples with only the welding intensity in the whole experimental process of testing for tensile strength having the same operational condition. Figure 3a,b and Table 4 show the results of the tensile tests. As the results in Table 4 show, the maximum strength of the welds was achieved at 225.9 MPa, and the average was 208.5 MPa. The average value of the specimens was higher than the tensile strength of the welding wire ER4043 (165 MPa), as reported in [14] at 26.4%, and this average value was equivalent to the 85% tensile strength of the A5052 aluminum alloy, as reported in [10]. The results indicated that good quality joints were obtained. In the tensile tests all specimens were failed at the brazing steel interface when they reached a maximum value as shown in Figure 3c. The results of the tensile tests for the seven welded specimens are presented in Table 4.

**Figure 4:**
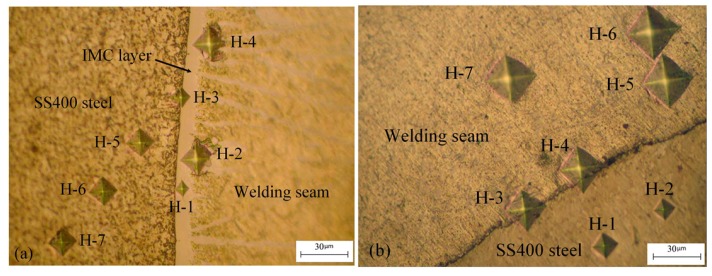
(**a**) Microstructure hardness is tested in intermetallic layer zone A in Figure 2; and (**b**) without IMC layer zone B in Figure 2.

Figure 4 shows the results of the microstructural hardness tests in the IMC layer zone. At the intermetallic layer zone, the maximum testing result was 850 HV, the minimum was 182 HV, and the average was 346.43 HV. Without the IMC layer zone, the maximum value measured was 291 HV, the minimum value was 85.3 HV, and the average was 117.85 HV. The hardness average values at the IMC layer zone and without the IMC layer zone in this research were higher than that of the welding wire ER4043 (56–64 HV), as compared to the hardness reported in [14], with evidence of the low dynamic load capacity and brittleness of the welds. Because the IMC layer was not good for the dynamic load and the load of the welds, it was necessary to find a way to reduce the thickness of the IMC layer as much as possible in order to increase the load capacity and the strength of the welding joints. The specific values of the tested positions for the hardness of the weld microstructures with the IMC layer and without the IMC layer zone are shown in Table 5.

**Table 4:** Results of tensile test of seven specimens’ welded A5052 aluminum alloys/SS400 steel.

Specimens	P1	P2	P3	P4	P5	P6	P7	Average	ER4043	A5052
Values (MPa)	206.5	203	195.5	220	225.9	215	193.5	208.5	165	250

**Table 5:** Results of microhardness test at seven points with an IMC layer and without IMC layer.

Specimens	H-1	H-2	H-3	H-4	H-5	H-6	H-7	Average	ER4043
IMC layer (HV)	850	209	468	182	240	237	239	346.43	61
Without IMC layer (HV)	287	291	129	118	86.2	85.3	88.3	117.85	

**Figure 5:**
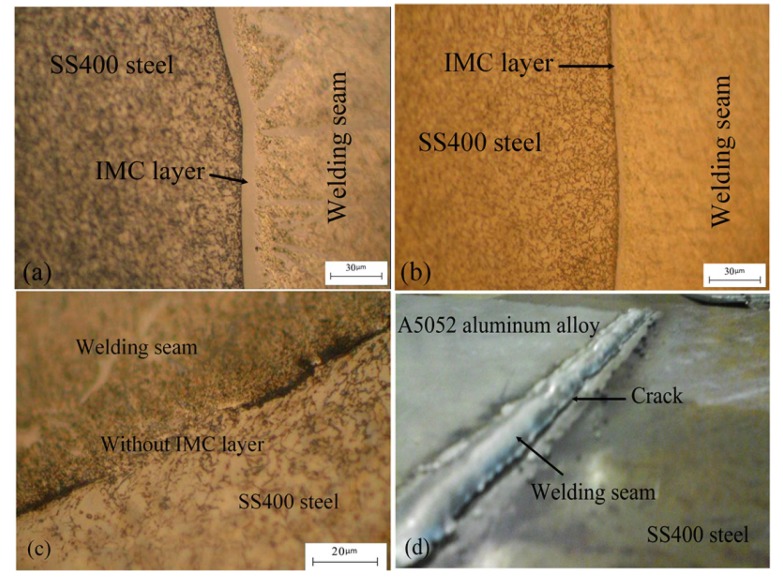
Microstructure of welded A5052 aluminum alloy/SS400 steel sheet for a welding intensity of 95 A and a welding speed of 3.5 to 4 mm/s: (**a**) IMC layer zone on the top side; (**b**) IMC layer zone on the bottom side; (**c**) without the IMC layer zone; and (**d**) cracking after welding.

For the microstructure of the welding seam and the SS400 steel plate, when welding A5052 aluminum alloy to steel, our study used a welding intensity of 95 A and a welding speed from 3.5 to 4 mm/s, as shown in Figure 5a. Figure 5b shows the results of the welding on the bottom side. A minimum IMC layer about 3 μm in thickness was produced with a suitable intensity and velocity; the welding seam was a seamless crystal lattice, which helped the weld to possess high mechanical properties. Figure 5a shows the welding of the top side; the two metal plates were at room temperature so the IMC layer here was thicker than on the bottom side. The bottom side was welded after the two metal plates had cooled. When welding the bottom side, because the first background layer was created, the Fe atoms at the solid-liquid interface reach a supersaturated concentration faster because of the current power from the welding process and the background of the top side. Furthermore, the first IMC layers were thin and stable so the flow of the metal liquid on the steel surface was better, as seen in Figure 5b. Other areas had better mechanical properties, especially near the welding area, some far from the center heat source and many parts without IMC, as seen in Figure 5c. As Figure 5d shows, an IMC layer appeared or did not appear, depending on the welding process used and the appropriate welding speed and intensity. The temperature of the steel sheets increased when the arc directly affected the surface of the steel plate during the welding process. Furthermore, on a thick IMC layer, cracks appeared between the surface of the SS400 steel sheets and the welding seam when a high welding intensity and low welding speed were used. We could see that choosing a suitable welding intensity has great influence on the fusion of Al base metal as well as the wettability and spreading ability of the weld metal; it can also lead to the quality change of welds, and as seen, affect the formation of the continuous surface appearance.

**Figure 6:**
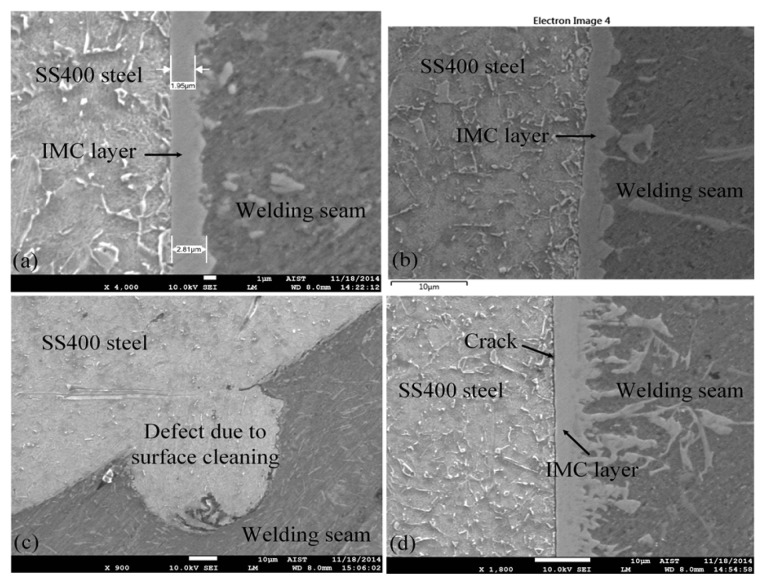
SEM microstructure at area between the welding seam and SS400 steel sheet: (**a**) IMC layer area; (**b**) without IMC layer area; (**c**) defects due to surface cleaning; and (**d**) microstructural crack on the surface of the steel sheet.

Figure 6 presents the morphology of the brazed interface layer between the welding seam metal and the SS400 steel. It can be seen that two main IMC layers formed along the brazed interface, and that a thin layer connected to the SS400 steel sheet surface; it includes the undulating surface structure in the welding seam metal and the serrated structure and rupture in the welding seam. The welding seam joint was formed between the base aluminum alloy and the welding wire in the welding process, and a thin IMC layer formed mainly along the brazed interface and the adjacent SS400 steel surface. The IMC layer was found to be about 1.95–5 μm thick between the welding seam and the SS400 steel surface after SEM testing. There were no defects at the welding seam, and the area had good mechanical properties, as shown in Figure 6a. With the 5 wt % of Si additions in the ER4043 welding wire, Si atoms inside the molten pool enrich the interface and often tend to aggregate to the interface because it can decrease the formation enthalpy of the IMC layer [15]. The IMC layer was thick and uneven as the welding was done by hand and the temperature in the area was uneven. Figure 6b shows the microstructure of the IMC between the welding seam and SS400 steel. A seamless metal structure with no defects has good mechanical properties, as found at the welding seam with length about 3–7 μm. It was clear that Si atoms appeared in the welding seam due to the use of ER4043 welding wire in the welding process. As the welding was done by hand, the temperatures of different areas were uneven, which made the IMC layer thick and uneven. Before welding, the edge of the SS400 steel plate was chamfered using a hand grinding machine and then cleaned using sandpaper. The cleaning process for the steel plate surface before welding was not good; this affected the welding quality and caused other defects, as seen in Figure 6c. Figure 6d shows the microstructural cracks on the surface steel sheet because of the high welding intensity and low welding speed, which caused the steel sheet surface to overheat and cracks to appear along the welds.

**Figure 7:**
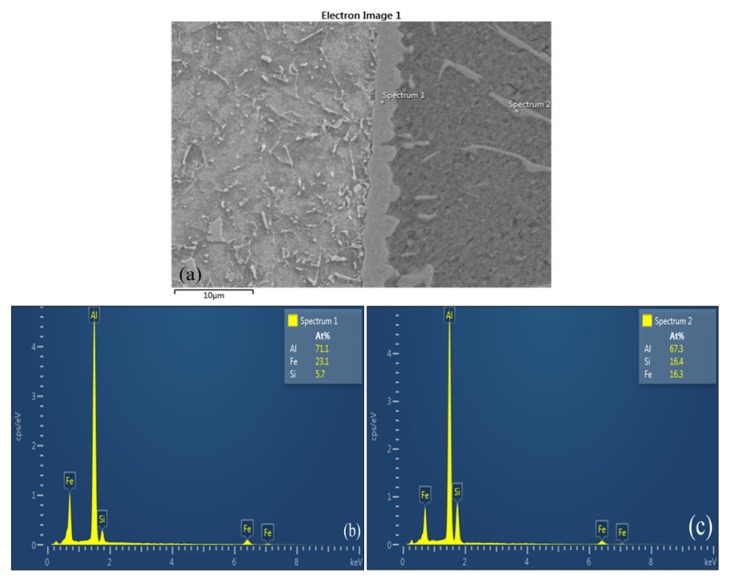
Energy dispersive X-ray spectrometer (EDS) analysis results of butt joint of A5052 aluminum alloy and SS400 steel: (**a**) Analysis positions; (**b**) Corresponding amounts at spectrum 1; (**c**) Corresponding amounts at spectrum 2.

According to the Fe-Al binary phase diagram reported in [16,17,18], there are five types of Fe-Al IMC phases, Fe_3_Al, FeAl, FeAl_2_, Fe_2_Al_5_ and FeAl_3_, which appear during the Fe/Al reaction process. The formation of a new phase is often determined by the thermodynamic conditions: the lower the free energy, the easier the phase forms during the process of multiphase formation. The interface between the steel and Fe_2_Al_5_ was smooth, and serrated shapes formed with the interface between the welding seam metals and FeAl_3_. Furthermore, the formation of new phases were often decided by the thermodynamic conditions and low the free energy. This leads to form process of multi-phase easy. Therefore, the formation of Fe-Al IMC layers could be predicted based on the thermodynamic data of the free energy. This has proved that the phase formed is Fe_4_Al_13_ during the chemical reaction between steel and aluminum. Figure 7 presents the metal components in the IMC area, at spectrum 1 in the middle of the IMC layer and at spectrum 2 in the welding seam, as determined using an energy dispersive X-ray spectrometer (EDS). The results also showed some diffusion in the welding process. Figure 7b,c present the results of the chemical composition analysis of the IMC. The elements at position number 1 were Al = 71.1%, Fe = 23.1%, and Si = 5.7%; the elements at position number 2 were Al = 67.3%, Si = 16.4%, and Fe = 16.3%. The results showed that the IMC layer consisted of three elements, Al, Fe and Si, signifying that this IMC layer was likely composed of a three-chemical-element Fe_2_Al_8_Si phase, and not a two-chemical-element Fe_4_Al_13_ phase.

**Figure 8:**
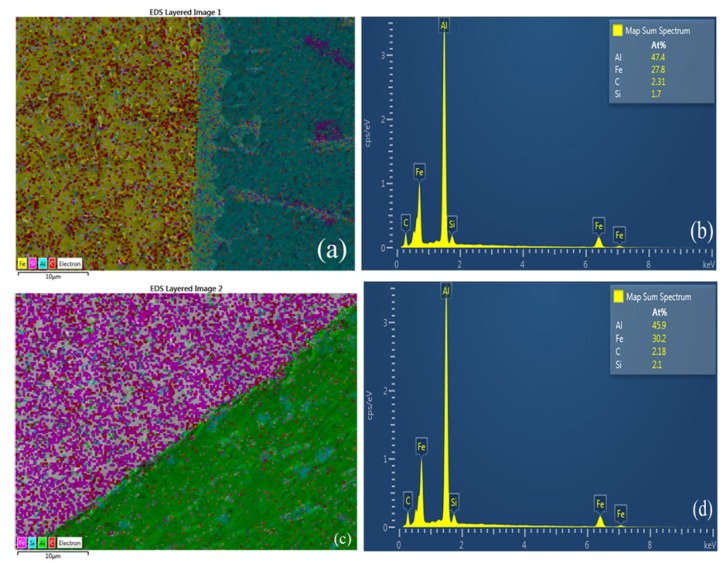
X-ray element mapping of butt joint of A5052 aluminum alloy and SS400 steel: (**a**) IMC layer zone; (**b**) Corresponding amounts at IMC layer; (**c**) Without IMC layer; (**d**) Corresponding amounts without IMC layer.

The X-ray mapping of the elements at the intermetallic layer zone between the SS400 steel and the welding seam at the IMC layer zone and without the IMC layer is provided in Figure 8a. Some atoms diffused during the welding process. Figure 8b shows the elements with their corresponding amounts which appeared at the welding seam area: Al (47.4%), Fe (27.8%), C (2.31%), and Si (1.7%). Figure 8c shows the results of the scanning of the weld without the intermetallic layer. At the investigated position, the four elements and corresponding amounts were Al (45.9%), Fe (30.2%), C (2.18%), and Si (2.1%), as shown in Figure 8d.

**Figure 9:**
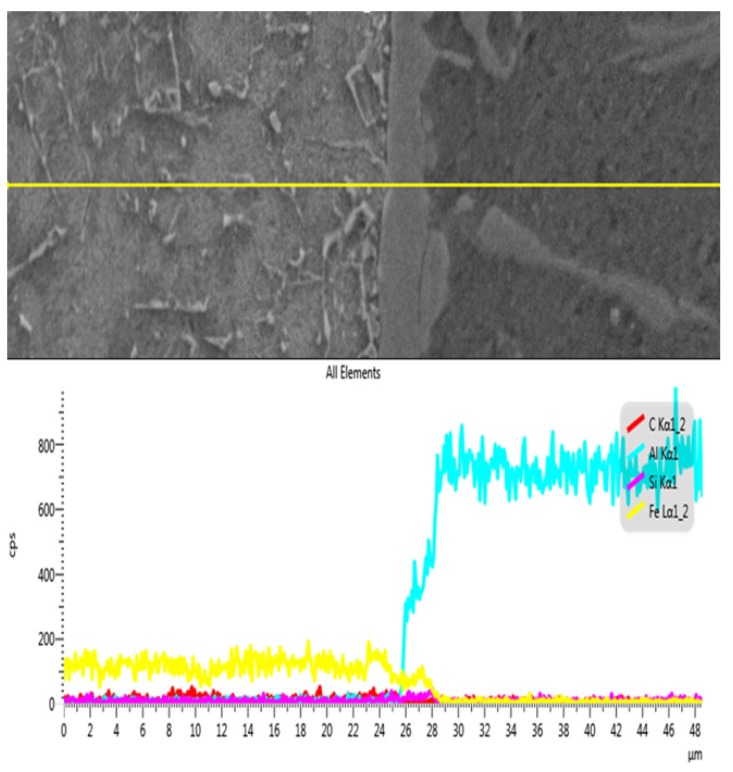
EDS results between the welding seam and SS400 steel interface layer.

Figure 9 shows the results of the EDS analysis between the welding seam and SS400 steel interface layer obtained in line scanning mode. Three elements, Al, Si and Fe, were found in the IMC layer, so this IMC layer was formed by ternary Al-Fe-Si IMCs. Especially at the IMC, the quantity of Si was higher than the other elements, which meant that Si atoms tended to appear in the area where there is a compound IMC layer to likely form a Fe_2_Al_8_Si phase.

## 4. Conclusions

The welding of two dissimilar metals, A5052 aluminum alloy and SS400 steel, was conducted using MIG with ER4043 filler metal and the new chamfering method. The tensile strength, microhardness, and microstructural characteristics were investigated. The conclusions drawn from this research can be summarized as follows:

- Welding dissimilar metals using the new chamfering method was successful on the front side and the bottom side. Because the SS400 steel had a higher melting temperature, it only melted a bit at the surface; the A5052 alloy had a lower melting temperature, so it melted completely.
- As the welding was done by hand, the thickness of the intermetallic layer was not uniform, ranging from 1.95–5 μm, and formed on the surface of the steel sheet. Minimizing the thickness of the intermetallic layer improved the quality of the welds. The SEM/EDS analysis showed the metallurgical interaction between the welding seam and SS400 steel.
- The average value of the tensile strength of the specimens was higher than the tensile strength of the welding wire ER4043, at 26.4%, and the average value was equivalent to 85% of the tensile strength of the A5052 aluminum alloy. The average microhardness value at the IMC layer zone and without the IMC layer zone in this research was higher than that of the welding wire ER4043 (56–64 HV), showing that the dynamic load capacity was low and the welds were brittle.
- Cracks, resulting from the use of high power in the welding process, were found between the welding seam and SS400 chamfered steel when the IMC layer was too thick.
